# Exploring the Proteomic Alterations from Untreated and Cryoablation and Irradiation Treated Giant Cell Tumors of Bone Using Liquid-Chromatography Tandem Mass Spectrometry

**DOI:** 10.3390/molecules25225355

**Published:** 2020-11-16

**Authors:** Rashmi Madda, Chao-Ming Chen, Cheng-Fong Chen, Jir-You Wang, Po-Kuei Wu, Wei-Ming Chen

**Affiliations:** 1 Department of Orthopedics & Traumatology, Taipei Veterans General Hospital; Taipei City 112, Taiwan; rashmi.akula86@gmail.com (R.M.); excelnova@gmail.com (C.-M.C.); cf_chen@vghtpe.gov.tw (C.-F.C.); yollywang@gmail.com (J.-Y.W.); wmchen@vghtpe.gov.tw (W.-M.C.); 2 Department of Orthopedics, Therapeutical and Musculoskeletal Tumor Research Center, Taipei Veterans General Hospital; Taipei City 112, Taiwan; 3 Orthopedic Department, School of Medicine, National Yang-Ming University; Taipei City 112, Taiwan; 4 Institute of Clinical Medicine, School of Medicine, National Yang-Ming University; Taipei City 112, Taiwan

**Keywords:** giant cell tumor of bone, liquid chromatography, mass spectrometry, biological samples, proteomic analysis, cryoablation, irradiation, matrix metalloproteinases, TGF-beta

## Abstract

Giant cell tumors of bone (GCT) are benign tumors that show a locally aggressive nature and affect bones’ architecture. Recently, cryoablation and irradiation treatments have shown promising results in GCT patients with faster recovery and less recurrence and metastasis. Therefore, it became a gold standard surgical treatment for patients. Hence, we have compared GCT-untreated, cryoablation, and irradiation-treated samples to identify protein alterations using high-frequency liquid chromatography-electrospray ionization tandem mass spectrometry (LC-ESI-MS/MS). Our label-free quantification analysis revealed a total of 107 proteins (*p* < 0.01) with 26 up-regulated (<2-folds to 5-fold), and 81 down-regulated (>0.1 to 0.5 folds) proteins were identified from GCT-untreated and treated groups. Based on pathway analysis, most of the identified up-regulated proteins involved in critical metabolic functions associated with tumor proliferation, angiogenesis, and metastasis. On the other hand, the down-regulated proteins involved in glycolysis, tumor microenvironment, and apoptosis. The observed higher expressions of matrix metalloproteinase 9 (MMP9) and TGF-beta in the GCT-untreated group associated with bones’ osteolytic process. Interestingly, both the proteins showed reduced expressions after cryoablation treatment, and contrast expressions identified in the irradiation treated group. Therefore, these expressions were confirmed by immunoblot analysis. In addition to these, several glycolytic enzymes, immune markers, extracellular matrix (ECM), and heat shock proteins showed adverse expressions in the GCT-untreated group were identified with favorable regulations after treatment. Therefore, the identified expression profiles will provide a better picture of treatment efficacy and effect on the molecular environment of GCT.

## 1. Introduction

Giant cell tumor (GCT) of bone is a benign tumor that shows locally aggressive metastasis with frequent recurrence [1]. These tumors grow at the ends of the long bones and create substantial disturbance in the local bony architecture, which causes severe effects on the peri-articular locations [2]. Majority of GCTs identified at the lower end of the femur or upper end of the tibia adjacent to the knee joint [2]. Their location, progression, and osteolytic nature quickly lead to a disabling functional impact, especially for the younger patients that are typically affected [3]. As GCTs are considered benign, and recurrence and metastasis rate occurs, 1–9% of patients show aggressive tumor growth [4]. These tumors are typically observed in young adults and especially common in females [2]. Due to their aggressive behavior, they can destroy the surrounding bone and cause serious complications. So far, the only treatment option available is surgery to remove the tumor and prevent further damage to the bone and the near affected joint [5]. GCTs formed by a network of a spindle or round-shaped mononuclear histolytic cells, or multinuclear giant cells were observed histologically [6]. Thus, physicians recommend intralesional curettage as a safe treatment option for maintaining the bone function; however, it has a high risk of local recurrence and metastasis [7]. Thus, several local adjuvant therapies were employed, such as phenol, alcohol, and cryoablation [8,9]. Among these adjuvant treatments, cryoablation/cryosurgery, become a gold standard procedure in treating GCTs due to less recurrence and metastasis [10,11]. 

The basic principle of cryoablation is to use extreme cold to induce necrosis, and the following ablative effect causes the tumor cells death [12]. As we know, liquid nitrogen at −197 °C is an effective cryogenic agent used for either tissue preservation or destruction. The rapidly freezing and slowly thawing phenomena of cryoablation cause tissue destruction [12]. Therefore, it has been operating as a primary salvage therapy for preserving the fiber framework of bones [9,11]. Especially for bone tumors, cryoablation is an effective treatment that kills tumor cells on the bone region excellently [10,13]. Moreover, our recent study on GCT cryoablation treated patients observed less recurrence and metastasis when compared with radiotherapy treated group [9,14]. However, despite its clinical significance, the exact mechanisms and pathways involved in the efficacy of cryotherapy treatment in GCT remain unclear. In addition to this, our recent comparative proteomic findings on Osteosarcoma (OGS)-untreated and treated with cryotherapy and irradiation revealed several significantly altered protein expressions that are involved in the healing, repair, and bone remodeling process [15]. This could be one of the reasons behind OGS patient’s low recurrence and metastasis. Therefore, in this study, we focus on GCTs to identify potential protein/molecular expressions that are differentially expressed after cryotherapy and irradiation treatment compared to the untreated. To further understand the biological and molecular functions of the identified proteins from proteomic analysis, we further performed Gene Ontology (GO) and protein-protein interaction networks (PPI) analysis. Furthermore, the confirmation of identified selected protein expressions from LC-MS/MS analysis was verified using immunoblot examination. Therefore, in this study, we will obtain a deeper and clear understanding of differential regulations of proteins during cryoablation and irradiation treatments on GCT that may shed some light on therapeutic targets. 

## 2. Results

### 2.1. Protein Expression Profiles from GCT-Untreated/Control, and Cryoablation and Irradiation Treated Groups 

The identified proteins from LC-ESI-MS/MS were compared among GCT-untreated/control and GCT-treated groups using label-free quantification based on two unique peptides with a false discovery rate (FDR) of 0.1%. From the three groups, a total of 1777 proteins were identified. Among these proteins, 656 were identified from the GCT-untreated/control group (Table S1) with a fold change of <2 to >0.5 (FDR: 0.1%; *p* < 0.05). On the other hand, 534 proteins (*p* < 0.01, FDR: 0.1%) were identified from GCT- cryoablation-treated (Table S2), and 548 proteins (*p* < 0.03, FDR: 0.1%) from irradiation treated (Table S3) groups. Among these proteins, 492 were commonly identified in GCT-untreated/control to the cryoablation and irradiation treated groups. All the identified proteins from the GCT-untreated/control and treated groups using electrospray ionization liquid chromatography and tandem mass spectrometry (LC-ESI-MS/MS) were found in supplementary file 1 (Table S4).

**Figure 1:**
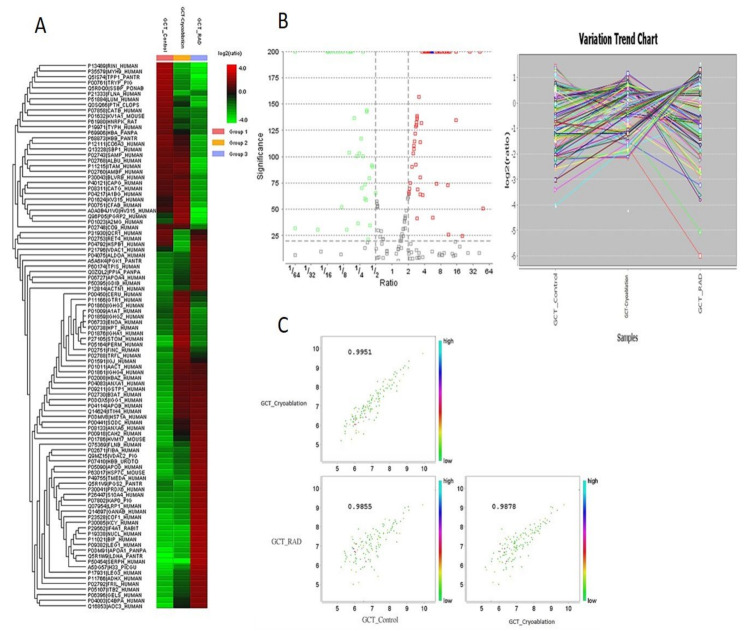
Label-free quantification analysis of 107 differentially expressed proteins from comparative giant cell tumor of bone samples. (**A**) Heat maps were generated using Peaks X proteomic software that is showing various classes of protein expression profiles of giant cell tumors of bone (GCT) compared among untreated and cryoablation and irradiation treated. The log2 ratios of the abundance of each sample relative to the average abundance. (**B**) Volcano plot analysis showing the up-regulated proteins in red color and down-regulated proteins in green and the similarly expressed proteins in gray squares. (**C**) Identified proteins correlation among three groups for the protein names see Table 1 and Table 2.

For the screening of differentially expressed proteins (DEPs) from the treated and untreated GCT//control samples, we choose the proteins expressed with at least 2-fold increased expressions, or less than 0.5-fold difference with a significant score of ≥50 were considered as differentially regulated. The identified proteins from this analysis and their names, accession numbers, and abundances were obtained from the Swiss-Prot database. Volcano plot analysis was performed with an FDR of <0.1 at a 95% confidence range to confirm the identified protein’s significance among GCT-untreated//control vs. GCT-cryoablation treated and GCT-irradiation (GCT-RAD) treated groups. We have employed a strict filtering to eliminate the redundant proteins from our analysis using a high significance score of 200 in the PEAKS X software. The differentially altered proteins with <2 folds to >0.5 folds among the tested samples were generated in a heat map illustrated in Figure 1.

**Figure 2:**
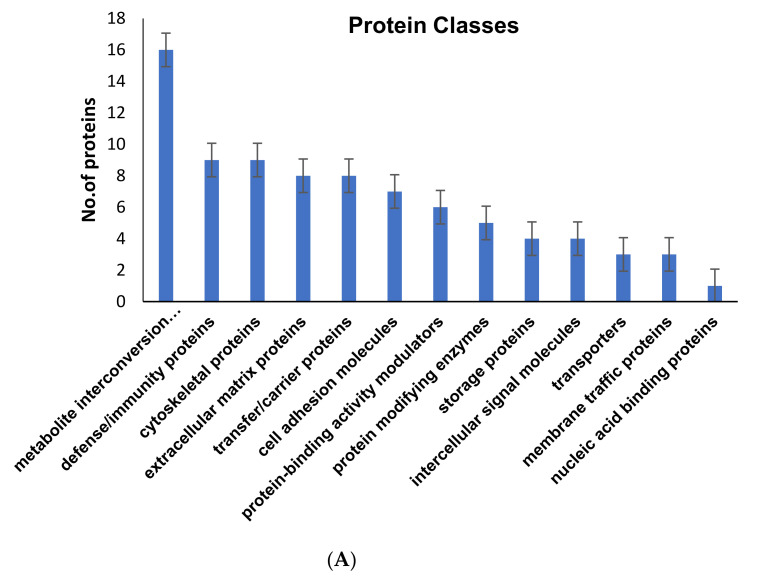

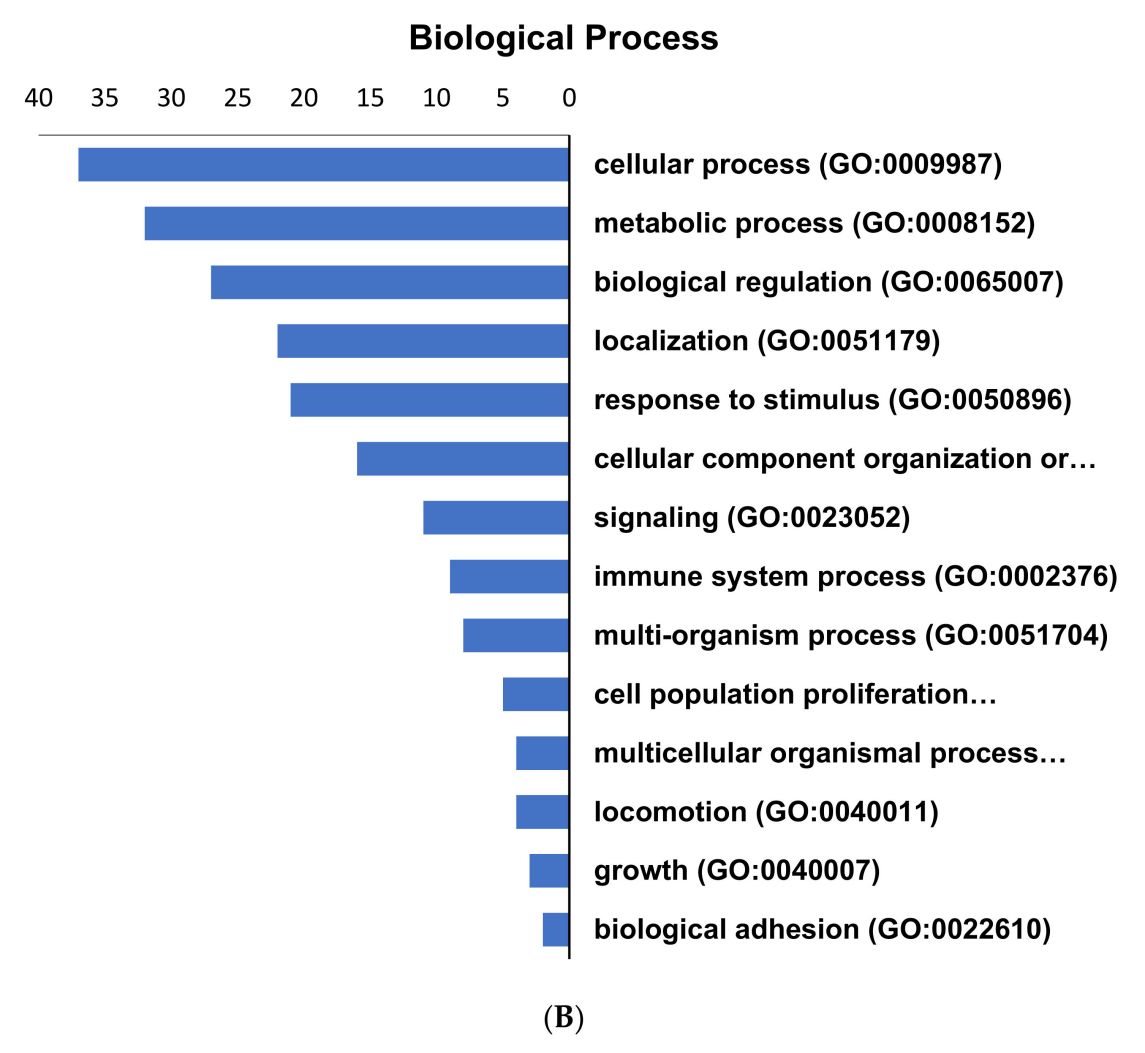
Gene ontology (GO) functional enrichment analysis of the identified proteins from GCT analysis performed by DAVID v6.8.24 with the GO terms of FDR < 0.01 (**A**) Protein Classes (**B**) Biological process.

To know the abundances of these identified DEPs from three groups, we used label-free quantification which demonstrated that 107 proteins were differentially expressed among GCT-untreated//control and treated groups with a significance of *p* < 0.01. Among these 26 proteins (*p* < 0.01; FDR < 0.1) were up-regulated with a fold change of <2-folds, and 81 proteins (*p* < 0.02; FDR < 0.1) were down-regulated with a fold change of >0.5 folds in GCT-untreated/control group, whereas 50 proteins (*p* < 0.01; FDR < 0.1) were up-regulated with a fold change of <2 to 5 and 45 proteins (*p* < 0.01; FDR < 0.1) were down-regulated with a fold change of >0.1 to 0.5 folds in GCT-cryoablation treated compared to GCT-untreated/control. On the other hand, 58 proteins were increased with a fold change of <2-fold, and 37 proteins (*p* < 0.031; FDR < 0.1) were dysregulated with a fold change of >0.1 to 0.5 folds in GCT-irradiation treated groups. These altered proteins from GCT were typically involved in various metabolic functions, including cytoskeletal integrity, oxidative stress, transcriptional regulation, apoptosis, angiogenesis, metastasis, and tumor microenvironment. Based on Gene Ontology (GO) analysis that was employed by PANTHER and DAVID revealed that the identified proteins were strongly enriched with various biological processes illustrated in Figure 2A. Based on GO enrichment analysis majority of the proteins were primarily involved in cellular and metabolic processes (37 proteins), translation (14 proteins), cytoskeletal organization (8 proteins), regulation of vesicle-mediated transport (12 proteins), biological regulation (30 proteins), cellular component organization (19 proteins), immune system process (15 proteins), and signaling (13 proteins) were illustrated in Figure 2B. These proteins are also involved in regulating various molecular functions such as translation, transcription, transportation, glycolysis, catalytic activity, RNA, and cytoskeletal protein binding. 

### 2.2. Exploring the Altered Protein Expressions in GCT-Treated and Untreated/Control Tumors

A total of 26 proteins consistently up-regulated with <2 to 5-fold expressions in GCT-untreated/control group compared to the treated. Among these proteins, Matrix metallopeptidase 9 (MMP9), TGF-beta, Cytochrome b-c1 complex subunit 1, Cathepsin K, Tripeptidyl-peptidase, Serum amyloid *p*-component, Single-stranded DNA-binding protein, Collagen alpha-3(VI) chain, Fas-binding factor, Integrin alpha-M, Heat shock protein beta-1, etc., increased expressions. demonstrating adverse effect that triggers various molecular events inside the cells which may lead to tumor proliferation. Moreover, these are primarily involved in numerous crucial metabolic and biological processes such as translation, inflammation, angiogenesis, integrin signaling, and apoptosis. Amongst 26 up-regulated proteins, 13 showed down-regulated expressions after cryoablation treatment, and 16 proteins showed dysregulated expressions after irradiation treatment. 

**Figure 3:**
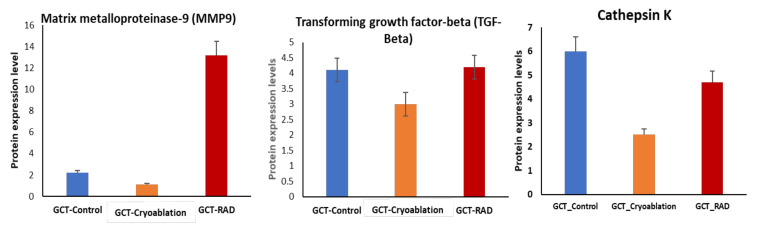
Differences in protein abundance of MMP9, TGF-Beta, Cathepsin K between GCT-untreated/GCT-Control (in blue), GCT-cryoablation treated (orange) and GCT-irradiation treated/GCT-RAD (in red). The higher expressions in GCT-untreated/control was showed reduced expressions after cryoablation treatment were under positive selection if they exhibited *p* < 0.01 and FDR > 0.01. *p* values represented in the figure corresponds to the two-tailed Student’s *t*-test. Error bars denote mean ± S.E.M.

Especially, MMP9, TGF-beta, and Cathepsin K were identified with <2 folds of up-regulated expressions in the GCT-untreated/control group showed decreased expressions in the GCT cryoablation treated group in Figure 3. It indicates, to some extent; there is a drastic change occurred inside the tumor cells before and after the treatments. Therefore, the rapid freezing and slow thawing phenomena of cryoablation not only effectively kills the tumor cells and also efficiently reduce the unfavorably regulating protein expressions—the complete list of up-regulated proteins from GCT-untreated/control Vs. GCT-Cryoablation and irradiation treated were listed in Table 1.

**Figure 4:**
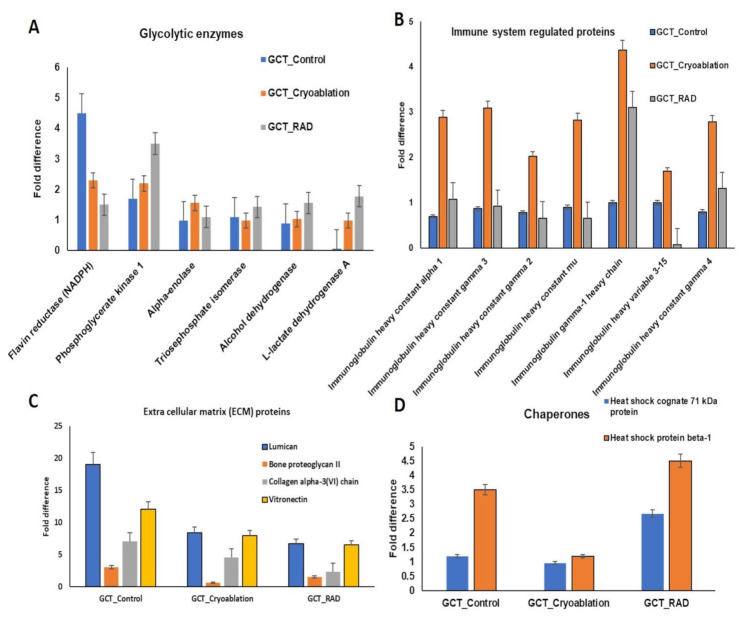
(**A**) Glycolytic enzymes, (**B**) Immune system proteins (**C**) Extra Cellular Matrix (ECM) proteins (**D**) Molecular Chaperons proteins that were significantly down-regulated from GCT-untreated/control compared to GCT-cryoablation and GCT-irradiation/GCT-RAD treated were identified using liquid chromatography tandem mass spectrometry were quantified using label-free quantification, after cryoablation and irradiation treatment the proteins were positively regulated *p* < 0.01 and FDR > 0.01. *p* values represented in the figure corresponds to the two-tailed Student’s *t*-test. Error bars denote mean ± S.E.M.

On the other hand, a total of 81 proteins showed significantly decreased expressions with <−0.1 to 0.5-folds in the GCT-untreated/control group. Among these, after cryoablation 39 proteins, and 55 proteins after irradiation treatment were up-regulated. From our gene ontology (GO) analysis, the identified up and down-regulated proteins of the untreated/control GCT group are classified as calcium-binding, cytoskeletal organization, defense/immune system regulation, extracellular membrane (ECM), acute phase proteins, immune system, and intracellular signaling molecules (Figure 4). In addition to these, several immune system-related proteins, metabolite repairing, and protein modifying enzymes such as glyceraldehyde-3-phosphate dehydrogenase, alcohol dehydrogenase, fructose-bisphosphate aldolase A, alpha-enolase, triosephosphate isomerase, UMP-CMP kinase, neutral alpha-glucosidase AB, myeloperoxidase and superoxide dismutase, etc., showed drastically decreased regulations in GCT-untreated/control group (Figure 4A, B). These dysregulated expressions may trigger or suppresses essential metabolic functions inside the tumor cells. Furthermore, the dysregulated expressions of metabolite repair enzymes contribute to causing lethal diseases in humans.

In addition to these, several essential ECM proteins, including lumican, vintronectin, collagen alpha III, etc., also reduced their expressions in the GCT-untreated/control group were shown in Figure 4C. Furthermore, the molecular chaperon heat shock protein 70 was down-regulated in the untreated/control group showed increased expressions after the cryoablation treatment of GCT. Our comparative proteomic analysis of GCT-untreated/control and GCT-cryoablation and irradiation treated revealed that both the treatments were efficient in regulating the adversely expressing proteins of GCT. Therefore, the identified increased expressions after cryoablation and irradiation treatments show the treatment response on tumors. The complete list of down-regulated expressions of GCT is listed in Table 2. 

### 2.3. Pathways Regulated by the Altered Proteins from GCT

**Figure 5:**
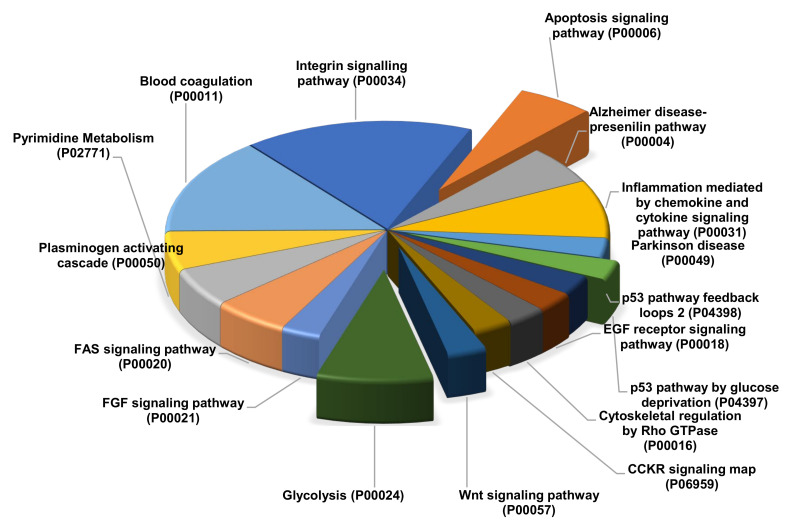
Gene Ontology (GO) pathway analysis using KEGG, PANTHER and DAVID v6.8.24 with FDR < 0.01 for the altered expressions of proteins from GCT comparative proteomic analysis involved into various critical pathways.

The identified differentially regulated proteins from this comparative proteomic study of GCT-untreated/control and treated were strongly involved in some potential pathways including angiogenesis (1.5%), apoptosis signaling (6%), CCKR signaling (2%), Integrin signaling (10.4%), Glycolysis (7.5%), cytoskeletal regulation by Rho GTPase (4%), FAS signaling (3%), p38 MAK pathway (3%), VEGF signaling (5%), etc., were showed in Figure 5.

### 2.4. Protein-Protein Interaction Networks (PPI)

**Figure 6:**
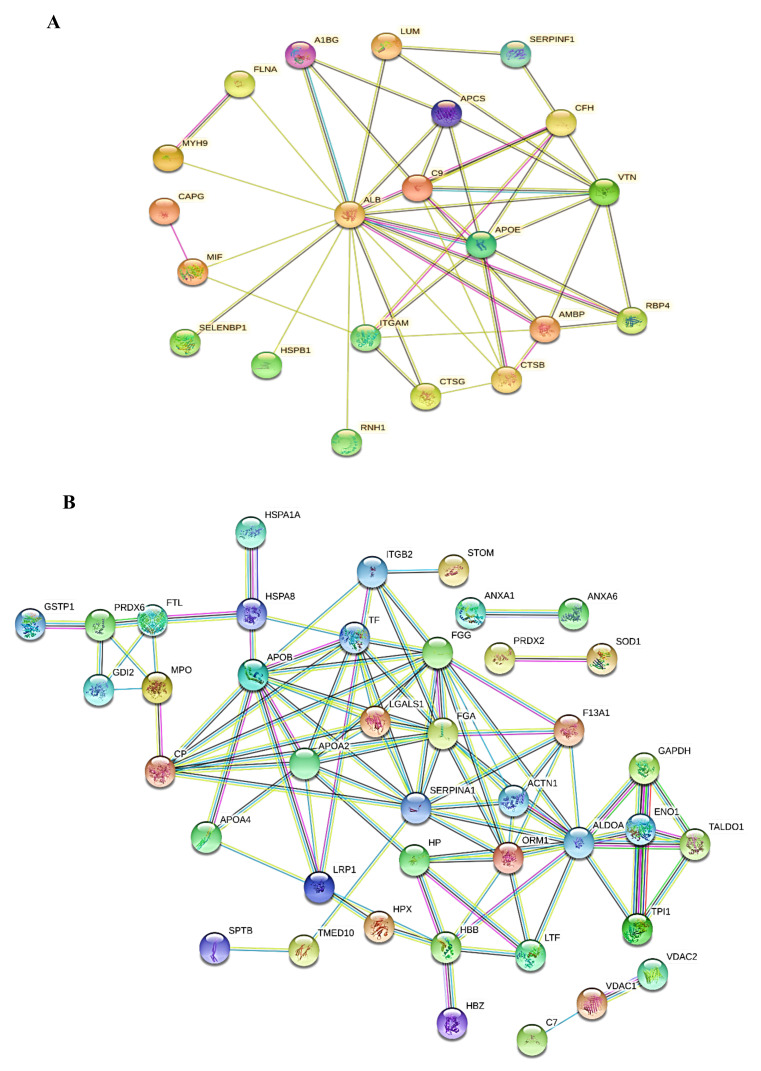
(**A**) Protein-Protein Interactions (PPI) of the up-regulated proteins of GCT untreated/control compared to the cryoablation and irradiation treated groups. (**B**) Down-regulated proteins from comparative analysis of GCT was performed by STRING v11.25. The proteins nodes were tightly networked represents the strong interaction among the proteins. The analysis was performed at highest confidence interaction score of 0.90. For protein full names, see Table 2.

To understand the key signaling networks of the identified proteins that are associated with cryoablation and irradiation treatment, we used STRING protein-protein interaction (PPI) network analysis. From our observations, the up-regulated proteins of GCT-untreated/control were tightly networked and negatively regulated numerous metabolic functions including cell migration, cell death, and regulation of angiogenesis. In addition to these, some proteins have closely interacted with the other proteins that may play an important role in negatively regulating the intrinsic apoptosis signaling pathway. This tightly interacted network map of the up-regulated proteins was shown in Figure 6A. On the other hand, the down-regulated proteins from GCT-untreated/control were strongly and tightly interacted and regulated several key metabolic processes such as glycolysis, negative regulation of apoptosis signaling, cell proliferation, and acute-phase inflammatory responses. The PPI network of down-regulated proteins were presented in Figure 6B.

### 2.5. Validation of MMP-9 and TGF-Beta Protein Expression in GCT

**Figure 7:**
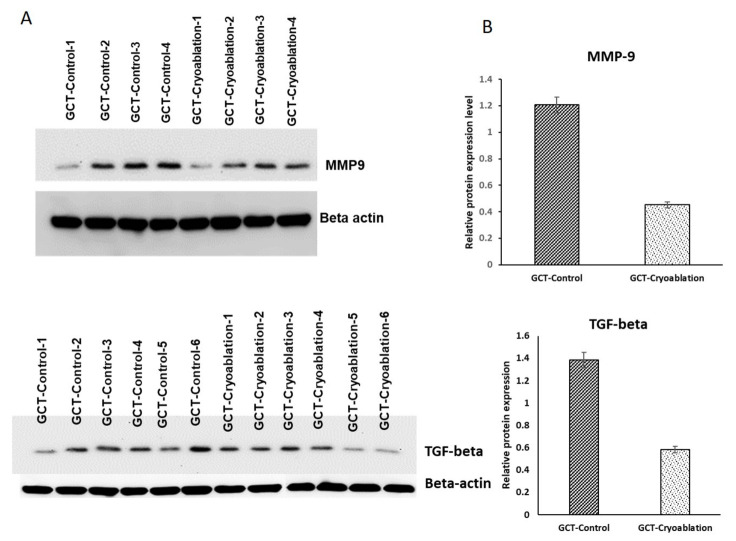
Validation analysis of the selected proteins from the giant cell tumor of bone profiling (**A**) MMP and TGF-beta immunoblot analysis. Both proteins increased expressions were identified in GCT-untreated/control showed reduced expressions after cryoablation treatment of GCT compared to GCT-untreated (GCT-control). (**B**) Bar charts representing the quantification data of overexpression’s of MMP9, TGF-beta were significantly expressed after cryoablation (*p*-value < 0.01; one-tailed Student’s *t*-test). Error bars of the treated samples denote mean ± S.D.

To confirm the identified up-regulated expressions of MMP 9 and TGF-beta from the GCT-untreated/control group a validation analysis was performed using a new set of GCT samples to make sure our results are accurate. The immunoblot analysis revealed both the proteins showed higher expressions in the untreated/control GCT group and down-regulated expressions in the cryoablation treated group. On the other hand, both the protein expressions were not changed in the irradiation treated GCT group were shown in Figure 7. These results revealed that MMP9 and TGF-beta expressions were remarkably correlated and consistent with the mass spectrometric identifications suggesting that cryoablation treatment has efficiently decreased the expression of MMP-9 and TGF-beta in GCT. However, irradiation treatment is not effective in reducing the higher expressions.

## 3. Discussion

The therapeutic effect of cryoablation and irradiation treatment on GCT of bones revealed that both the treatments were efficient and effective in killing the tumor cells and also regulate various metabolic events inside the cells. Several studies on GCT demonstrated the proteomic alterations of either untreated or treated using protein inhibitors such as denosumab [16,17]. However, no study discussed proteomic alterations after cryoablation and irradiation on GCT tumors. Therefore, we aimed to find the altered proteins from GCT untreated/control and treated tumors using mass spectrometry. Besides, our recent comparative proteomic study on Osteosarcoma (OGS) before and after cryoablation and irradiation treatment has shown various differential protein expressions that play an important role in regulating critical metabolic pathways, cell proliferation, signaling, apoptosis, tumor microenvironment, recovery, and healing. In addition to this, in our clinical practice, cryoablation and irradiation treated GCT patients showed less recurrence and metastasis. Thus, we continue our quest to find the altered protein expressions from GCT patients that may play an essential role in the tumor microenvironment, recurrence, and metastasis.

This study revealed that drastic biological and molecular alterations have occurred in GCT tumors after cryoablation and irradiation treatment. The identified higher expressions of proteins from GCT-untreated/control were regulated positively after the cryoablation and irradiation treatment that may play an important role in the recovery and healing process. Our label-free quantification analysis revealed that 107 proteins were differentially regulated after cryoablation and irradiation treatment. Among these, 26 proteins were up-regulated, and 81 proteins were down-regulated in the GCT-untreated group. Most of the up-regulated proteins from GCT-untreated/control were involved in various key signaling and metabolic pathways related to tumor cell growth and proliferation. Interestingly, most of the up-regulated protein expressions from the GCT-untreated/control group were significantly reduced after cryoablation and irradiation treatment, demonstrating that both treatments effectively regulate the adversely responding proteins in GCT.

From the up-regulated proteins of GCT-untreated/control, it is interesting to study matrix metalloproteinases-9 (MMP9) and TGF-Beta that showed reduced expressions after cryoablation treatment. From our analysis, both the proteins were continually showed increased expressions in the untreated-GCT group. Several studies demonstrated that in different tumor tissues, TGF-beta could down-regulate the levels of MMP9 [18]. As we know that MMP9 is one of the MMPs family protein that prominently degrades different components of the extracellular matrix [19,20]. Mounting evidence illustrated that MMP-9 (gelatinase B) might play a significant role in GCT tumor progression and invasion [21]. It is also involved in bone matrix destruction and osteolysis by degrading type-I collagen [22]. MMP9 is also associated with osteoclast differentiation by activating downstream signaling [23]. On the other hand, the elevated levels of TGF-beta from the GCT-untreated/control group indicates the possibility of cell migration [24]. Therefore, the reduced expressions after cryoablation treatment depict the treatment is efficacy in controlling the migration of cells that may eventually reduce the chances of metastasis. Due to MMP 9 and TGF beta’s prominent role in giant cell tumors of bone, we further analyzed these two proteins using western blot analysis and confirmed the proteomic expressions in a new set of GCT samples.

Another vital protein highly expressed in the untreated/control GCT group is Cathepsin K, a unique collagenase protein primarily observed in osteoclasts. It is critical for collagen matrix degradation, bone remodeling, and osteolysis in GCT [25]. Previous studies reported the higher expressions of Cathepsin K in osteoclasts, critically involved in bone homeostasis associated with bone turnover and loss of bone [26]. Our findings from the cryoablation-treated and irradiation treated groups were observed with reduced cathepsin K expressions indicating that no bone degradation was observed from both the treatments.

In addition to this, our analysis also identified several important ECM proteins such as lumican (LUM), and vimentin showed up-regulated expressions in the GCT-untreated/control group, demonstrating the proliferative activity [19]. The up-regulated expressions were reduced after cryoablation treatment. In addition to these, the molecular chaperons showed contrast expressions such as heat shock protein 70 (HSP 70) identified with higher expressions, and heat shock protein beta 1 (HSPB1) showed reduced expressions in the GCT-untreated/control group indicating tumor recurrence [27]. Interestingly, HSP 70 expressions were reduced by cryoablation but not by irradiation. It might be one of the reasons GCT-Cryoablation treated patients showed no recurrence. On the other hand, after irradiation treatment, more than 4-fold higher expressions of HSP 70 were identified in GCT. Thus, cryoablation treatment is more effective than irradiation in terms of tumor recurrence potential.

This study also identified up-regulated expressions of some crucial proteins, including thymidine phosphorylase (TP), myosin 9, ribonuclease inhibitor, pigment epithelium-derived factor (PEDF) in the GCT-untreated/control group. Previous studies reported higher expressions of these proteins are often identified in tumors that have a crucial role in angiogenesis, invasion, and tumorigenesis [28,29,30]. On the other hand, these proteins were downregulated after cryoablation and irradiation, demonstrating treatment efficacy in lowering the adversely expressing proteins after tumor cells’ death.

In addition to these, most of the key enzymes that play an essential role in glycolysis and molecular cell-signaling showed remarkably down-regulated expressions in GCT-untreated/control, indicating glycolysis has been critically affected due to the aggressive nature of the tumor. It has been hypothesized that cancer cells quickly adapt to a mechanism that provides biosynthetic requirements for the rapid proliferation of cells causing alterations of glycolytic enzymes [31]. Hence, the identified dysregulated protein expressions indicate that there could be a Warburg effect that occurred inside the tumor cells. Especially from our evaluations, we have identified several important proteins, including phosphoglycerate kinase 1 (PGK1), lactate dehydrogenase (LDHA), aldolase A (ALDOA), glucose transporter member 1 (GLUT1), alpha-enolase, etc., found to be altered. Mounting evidence demonstrated that the down-regulation of PGK1 is associated with a shorter survival rate [32,33,34,35]. And the altered expressions of LDHA, enolases play a potential role in tumorigenesis as tumor cells possess a higher metabolic rate than the normal tissues [32,36]. Moreover, the down-regulated expressions of Enolase alpha have been demonstrated in NSLC tumors that may play a vital role in lung tumorigenesis [36]. Therefore, the identified decreased enolase expressions of GCT-untreated/control tumors indicates the metastatic lung potential. Another important protein, GLUT 1that, plays a crucial role in glucose transport, and consumption also showed a drastic decrease in GCT-untreated/control tumors, demonstrates tumor cells rely on other glucose transporters to regulate their glycolytic pathway. Interestingly, after the treatment with cryoablation and irradiation, the levels were slightly increased, which determines the risk of metastasis may be lower after the treatment. On the other hand, irradiation showed higher expressions than expected, indicating chances of recurrence after irradiation. Therefore, cryoablation showed a more beneficial effect on GCT than irradiation. In addition to this, our clinical observations on GCT patients that are treated with cryoablation were also responded well with no recurrence and metastasis (data not shown).

Along with these, serum albumin, serotransferrins and some immune regulating proteins were also identified with deficient expressions in the untreated/control group of GCT. It illustrates a weakening of the immune system due to tumor cell proliferation. Surprisingly, after cryoablation, most immune system, proteins were significantly increased; on the other hand, contrast expressions were noted in the irradiation treated group. Therefore, after cryoablation, the immune markers will come into action to protect the cells [13,37]; this could be one of the great reasons that GCT-cryoablation treated patients observed faster recovery with no recurrence and metastasis.

## 4. Materials and Methods

### 4.1. Patients and Clinical Information

This study included a total of 12 giant cell tumor of bone (GCT) patients (male/female; 6/6; age ranging from 33–65 years). The GCT tissue samples were collected from Taipei Veterans General Hospital (VGH-TPE), Taiwan. Each collected tissue specimen of GCT patient was sectioned into three specimens for proteomic analysis the collected GCT samples were categorized into three different groups such as GCT-untreated/control (*n* = 12), GCT-cryoablation treated (*n* = 12), and GCT-untreated/control (*n* = 12) groups for comparative proteomic analysis. All samples were freshly collected from the operation theatre after the surgery without any chemotherapy, radiation and immunosuppressive medication were advised. The demographic and clinical features of the obtained samples were shown in Table 3. Diagnostic criteria of all the collected GCT patient samples were confirmed by a certified surgeon as well as a pathologist by the tissue biopsy examinations. The collected samples were stored at −80 °C for further analysis. This study confirmed and conducted all the materials and methodology according to the guidelines and regulations of IRB was approved by the institutional review board of VGH-TPE, Taiwan (IRB Approval No.2019-02-021A), and informed consent was obtained from all the patients.

### 4.2. Extraction of Protein from GCT Untreated/Control and Treated Samples Preparation

The 12 recruited GCT patient’s tumor tissues were categorized into three groups as cryoablation-treated irradiation-treated and untreated/control. To extract the protein from the treated the cryoablation/freezing group samples were subjected to liquid nitrogen freezing treatment for 15 min under complete sterilization conditions [38]. The treated GCT tissue samples were thawed at room temperature for 20–25 min then pulverized by mortar and pestle using liquid nitrogen. For irradiation treated protein extraction, the samples were treated under 15,000 gamma radiation and then extracted the protein from the treated samples. Both the treated and untreated/control samples protein was extracted using RIPA lysis buffer (50 mM Tris-HCl pH7.2, 150 Mm NaCl, 1% NP40, 0.1% SDS, 0.5% DOC, 1 mM PMSF, 25 mM MgCl_2_) (Sigma, St. Louis, MO, USA; R0278) supplemented with a phosphatase inhibitor cocktail (Thermo, Waltham, MA, USA; 78420) Then the samples were centrifuged at 13,000× *g* for 15 min. Then, the supernatant was separated into new tubes then the extracted purified protein from all the cryoablation treated GCT and untreated/control were subjected to total protein concentration determination assays such as BCA [39] and Bradford (Bio-Rad Laboratories, Hercules, CA, USA).

### 4.3. Protein Precipitation and In-Solution Digestion

For the comparative proteomic profiling of GCT, the treated and untreated/control samples were analyzed using LC-ESI-MS/MS technology. Protein samples from both the groups were subjected to precipitation using a fourfold volume of 100% ice-cold acetone and incubated overnight at −20 °C. After precipitation, the samples were centrifuged at 14,000× *g* for 10 min, and the pellets were dissolved in 100 µL of 50 mM NH_4_HCO_3_ with 6.5 M urea (0.1–1 µg/µL) followed by an in-solution digestion procedure illustrated by earlier groups [40]. Then, the samples were reduced using 100 mM DTT (Dithiothreitol) added to each solution to make the final DTT concentration 10 mM at 37 °C for 30–40 min followed by alkylation step with 200 mM IAA (Iodoacetamide) which is added to each solution to make the final IAA concentration 20 mM incubated in the dark at room temperature for 25–35 min, separately. For the digestion of proteins sequencing grade trypsin (0.2 µg/µL) was reconstituted or diluted in the resuspension buffer (50 mM acetic acid), (Promega, Madison, WI, USA; V5111) and added the trypsin solution to a final ratio of 1:50 (*w/w*, trypsin: protein) and incubated at 37 °C for 16–20 h. Later, to quench the reaction 2 µL of 50% formic acid (FA) was added to the protein solution and mixed briefly, and incubated for 10 min. After incubation, the digest was briefly vortexed and centrifuged then the supernatant containing peptides were collected followed by lyophilization and desalting using C18 zip-tip technique [41].

### 4.4. Nano UPLC and Mass Spectrometry Conditions

SYNAPT G2-Si (Waters Corporation, Milford, MA, USA) LC-HDMSE with Masslynx™ (version 4.1, SCN 851) was used to acquire the proteomic data from three groups of GCT samples. The instrument was operated in high-resolution mode with a power of at least 20,000 FWHM at *m*/*z* 785.8427 (doubly charged positive ions, Glu-fibrinopeptide B). 400 ng peptides were digested and reconstituted in 3% ACN (Acetonitrile) and 0.1% FA (Formic Acid). Then, by using the C18 reverse-phase column (1.7 µm × 75 µm × 250 mm) (Waters Corporation, Milford, MA, USA), the digested peptides were separated. For our analysis, binary solvent system contained 99.9% water and 0.1% FA was considered as mobile phase, and 99.9% ACN and 0.1% FA performed as mobile phase B. At a flow rate of 5 µL/min using a 5 µm symmetry C18 trapping column (internal diameter 180 mm, length 20 mm) (Waters Corporation, Milford, MA, USA) with 0.1% FA was executed for all the peptides which were primarily pre-concentrated and desalted online. The peptides were then eluted successfully at a flow rate of 300 n/L and a gradient of 2% to 40% for 120 min into the Nano-LockSpray ion source subsequently employed to each injection. After all the injections, the column was appropriately washed and equilibrated in ESI positive mode for all the samples. For the mass spectrometer calibration [Glu1] fibrinopeptide solution (300 fmol/μL) was carried through the NanoLockSpray source. For the accurate LC-MS/MS data MS/MS mode of acquisition with mass scan range from *m*/*z* 50 to 2000, with a capillary voltage of 2.8 kV, a source temperature of 100 °C and a cone voltage of 30 V were employed. For the comparative proteomic evaluations of GCT treated with cryoablation, irradiation and untreated/control samples were run in triplicates and the raw data was analyzed by ProteinLynx Global Server 4.2 software (PLGS: Waters Corporation, Milford, MA, USA) and quantified using Proteome discover and PEAKS X software’s for more accuracy of the protein identifications.

### 4.5. Protein Quantification

We tried to analyze GCT tissue samples for proteomic analysis using high-resolution electron spray ionization liquid chromatography and tandem mass spectrometry (LC-ESI-MS/MS) (Waters Corporation, Milford, MA, USA) analysis. Label-free quantification was performed for the proteins identified from our LC-ESI-MS/MS investigation using PEAKS Studio X (Bioinformatics Solutions Inc. Waterloo, ON, USA) [42,43]. Analyzed triplicate independent samples were compared among cryoablation and irradiation treated and untreated/control groups of GCTs. The obtained raw data files of the analyzed samples were imported from the mass spectrometry instrument and uploaded all the raw data files to the quantitative PEAKS software program. The identified proteins from the triplicate tested samples each spectrum and its interpretation along with the alignment of the ion chromatogram and retention times were studied. For better accuracy, the retention time was specified as 600 to 10,500 s. The protein identification from the raw data was performed the same as we described in our earlier study, an Uniprot’s reference database of Homo sapiens (release 03_2014)36 contained 20,272 entries were added and combined with a decoy database (the sequences were reversed) was used. For label-free quantification a set of parameters were specified as follows: digestion by trypsin, with 2 missed cleavages; precursor mass tolerance was 10 ppm; fragment mass tolerance: 5 ppm, minimum charge: 2, maximum charge: 3, carbamidomethylation, oxidation (M), and deamidated (N and Q) were specified as fixed and variable modifications. The estimated spectra were employed against the decoy database for determining the false-positive identification rate. To obtain precise identifications of the proteins from each sample the quantifications were evaluated by false discovery rate (FDR) [44] of <1%, with a peptide score of −10 log *p* ≥ 20 was employed.

To determine the relative protein and peptide abundance in the tested samples, peptide feature-based quantification was performed as explained in our earlier studies [45]. For the accurate identification of peptide intensity differences among two samples the peptide signal intensity is equivalent to the abundance of the peptides in the sample; thus, the peptide features were corresponding accurately. These parameters allow us to quantify the differences in peptide intensity between GCT treated and untreated/control samples efficiently. Next, among three analyzed runs, the extracted ion chromatograms (XICs) and the area under the curves (AUC) were measured and compared. The total cumulative peak area of the identified proteins was determined by choosing only the unique peptides that are specifically stipulated to the particular proteins that were chosen. FDR was calculated based on the target/decoy database as mentioned in the earlier studies, [45] and the >1% FDR peptides were chosen as true positive hits (considering the chance of getting one false positive in 20 observations). With this active feature-based quantitative approach the identified peptides with *p*-values < 0.05 and 0.01 that were identified in at least three observations from the GCT both the treated and untreated/control were compared and measured. To determine the significance of protein expressions between treated GCT and untreated/control samples was explained in Section 4.8. The obtained spectral datasets were quantified and normalized the data to get the abundance factor values (triplicate analysis of the LC-MS/MS were averaged). The differentially expressed proteins (DEPs) were identified among the two groups were generated in a heatmap by peaks X software which illustrates the protein expressions. An individual false detection (FDR) rate was used to minimize the false positives and excluded the proteins with *p* >0.05 from further analysis. Proteins with an XIC value lower than 100,000 and identified in only one of the three technical replicates were observed as absent (noise), and excluded from our study. Both the treated and untreated/control GCT sample’s technical replicates XIC values were averaged and quantified, and the ratios of GCT-untreated/GCT-treated with cryoablation and irradiation were employed to identify the differentially expressed proteins as down-regulated proteins with <0.3–0.5 folds. Upregulated proteins were denoted with GCT-untreated/GCT-treated with a fold change of <1.5 to 2.

### 4.6. Protein Identification

The altered proteins from this study were further analyzed for the protein identification analysis using Mascot Software (Matrix Science version 2.2, http://www.matrixscience.com) [46] search engine along with the UniProtKB database (UniProt release 2015-10) [47,48] and National Center for Biotechnology non-redundant (NCBInr) [49]. In order to screen the proteins precisely we choose the following options for the identification: digestion by trypsin with two missed cleavages and carbamidomethyl specified as constant modification, and oxidation (M) as variable modification. Mass tolerance of 50 ppm and 0.1 Da MS/MS. To eliminate the false identifications from the obtained data < 1% of FDR were selected. The proteins that are consistently identified based on the stated parameters from all the three technical replicates or at least two of the three analyses were selected for further evaluations. The identification of theoretical molecular mass (MW) and isoelectric point (pI) of the identified proteins were determined using the Mascot database.

### 4.7. Bioinformatics Analysis

To understand the identified proteins involvement in biological processes (BPs) and their molecular functions (MFs), along with the protein categories and cellular components (CCs) an international standardized gene function classification system of gene-ontology (GO) (http://www.geneontology.org/) [50], PANTHER version 7.1, and the DAVID (http://david.abcc.ncifcrf.gov/) (Database Annotation Visualization, and Integrated Discovery) [51,52] database for functional analysis were performed. To evaluate the protein-protein interactions (PPI) among the identified proteins from GCT-untreated/control vs. treated we further analyzed our results using STRING (Search Tool for the Retrieval of Interacting Genes/Proteins, Version 9.1) PPI networks (website: http://string-db.org/) and specified the high score of 0.09 along with the default parameters for the significant results. From our analysis we have gained a better understanding of the identified proteins and their biological context and involvement in various pathways that are playing a potential role in pathogenesis and diagnosis of chondrosarcoma.

### 4.8. Statistical Analysis

The protein profiles of GCT-untreated/control Vs. cryoablation and irradiation treated patients’ samples were analyzed in triplicates, and the variations in the percentage of volume and relative intensity were confirmed by statistical analysis. The spectral counting evaluations were carried out to understand the altered expressions of the proteins quantified using LC-ESI-MS/MS data. Each sample was evaluated in three technical replicates and the average of the obtained abundance spectra was calculated. The data are expressed as mean ± standard deviation (SD) was determined using analysis of variance (ANOVA) assessment, [53] and Mann-Whitney U-test was performed by SPSS statistical package (SPSS19, SPSS Ltd., Woking, Surrey, UK) for Windows. A probability value < 0.05 was considered as statistically significant and < 0.01 was considered as highly significant.

### 4.9. Western Blot Analysis:

Validation of the selected proteins was carried using western blotting analysis in a new set (*n* = 6) of GCT bone tissue samples. And grouped as post and pre-treated GCT specimens. Proteins were separated by SDS-PAGE on to an electro transferred PVDF membrane (Millipore Corporation, Bedford, MA, USA) at 100 V for 60 min. In a TTBS solution [0.2 M TRIS-HCl (pH 7.6), 1.37 M NaCl, 0.1%Tween-20] [54], the transferred protein membranes were immersed in 5% non-fat milk for 1hr at room temperature. The proteins were incubated with primary antibodies, Matrix metalloproteinase (MMP9) rabbit monoclonal antibody (catalog no. 3852S, 1:1000 dilution), protein TGF-beta rabbit mAb (catalog: SC-3711S 1:1000 dilution), beta-actin rabbit mAb (catalog no. ab8227, 1:1000 dilution) at 4 °C overnight. All the antibodies were purchased from Abcam (www.abcam.com) (Cambridge, UK). Then the membranes were washed and incubated in 5% non-fat milk in a TTBS solution for 3 h at room temperature and subjected to three 5 min rinses in a TTBS solution. Then incubated 1 h at room temperature with a horseradish peroxidase-conjugated goat anti-rabbit antibody (Zhongshan Golden Bridge Biotechnology Co., Ltd., Beijing, China; catalog no. 7074), and washed 3 times for 5 min rinses in a TTBS solution. The blot was developed with a Super ECL Plus kit (Applygen, Beijing, China), and the signal was exposed to an X-ray film. The images were scanned, and the intensity of each band was captured using an Image Master 2D Platinum version 5.0 (GE Healthcare Amersham Bioscience). Then, each band intensity that was consistently observed was standardized as a percentage of the total intensity, and as referred to a relative volume that represents the relative expression abundance of the identified proteins in the tested samples. To evaluate the protein expression stability relative expression abundance was used.

## 5. Conclusions

This study is the first comparative proteomic profiling analysis of GCT untreated/control vs. GCT-treated with cryoablation and irradiation using mass spectrometry. Our label-free quantifications identified significantly altered proteins among untreated/control and treated groups that are typically involve in various key signaling pathways. Those are tumorigenesis, apoptosis, glycolysis, TGF-beta signaling, and triggers various metabolic interactions in GCT. These comparative proteomic identifications revealed how cryoablation and irradiation regulate the protein expressions and play an essential role in recurrence and metastasis of GCT. From our observations, cryoablation treatment is more effective in killing 100% of tumor cells and positively regulating the proteins to minimize recurrence and metastatic potential in GCT.

### Limitations

Our study had some limitations: (1) For the best understanding of the molecular changes that occurred after cryoablation, and it would be good to study the proteomic changes in larger population. (2) Although we have illustrated various categories of proteins that may be potential markers of GCT, we validated only two markers by Western blotting due to the unavailability of expensive antibodies and limited samples. (3) Validating some of the potential markers of GCT from this study in biofluids like serum or plasma after cryoablation treated patients’ samples would be a good strategy for biomarker discovery.

## Figures and Tables

**Table 1:** Elevated levels of proteins identified from untreated//control giant cell tumor of bone compared to cryoablation and irradiation treated.

Accession	Protein Name ^a^	Significance	Coverage (%)	#Peptides	#Unique	GCT_Untreated//Control ^b^	GCT_Cryoablation ^b^	GCT_RAD	*p*-Value
FLNA_HUMAN	Filamin	200	12	7	6	1.763093	−1.42652	−3.1814	5.24 × 10^−6^
PDL1_HUMAN	Programmed cell death ligand 1	200	91	81	18	1.15715	−0.92406	−2.84759	0.0005
LUM_HUMAN	Lumican	200	21	2	1	1.060151	0.151582	−1.45149	0.001
ALBU_HUMAN	ALBUMIN	200	17	15	15	1.040816	0.673168	−1.83586	0.001
THRB_HUMAN	Prothrombin	200	11	6	4	0.911502	0.542668	−3.46715	0.04
CO6A3_HUMAN	Collagen alpha-3(VI) chain	200	61	20	1	0.789581	0.609516	−1.85642	0.01
BLVRB_HUMAN	Flavin reductase (NADPH)	200	14	8	5	0.796578	0.568552	−0.76413	0.04
SBP1_HUMAN	Methanethiol oxidase	200	15	9	3	0.691594	0.360094	−1.34739	0.00012
HBA_HUMAN	Hemoglobin subunit alpha	200	18	5	1	0.650356	0.159428	−0.585	0.04
HBB_HUMAN	Hemoglobin subunit beta	200	8	5	1	0.712071	0.219155	−0.34253	0.0023
VTNC_HUMAN	Vitronectin	200	18	5	1	0.659152	0.04037	−0.24675	0.004
MMP9_HUMAN	Matrix metalloproteinase 9	200	8	5	1	0.63959	−0.1886	−0.58489	0.01
CATB_HUMAN	Cathepsin B	200	15	5	1	0.626248	−0.6555	−0.93563	0.0007
SAMP_HUMAN	Serum amyloid P-component	200	8	5	1	0.454084	0.310969	−1.43804	0.02
ITAM_HUMAN	Integrin alpha-M	200	5	3	1	0.388519	0.57785	−2.65341	0.04
CSF1_HUMAN	Colony stimulating factor 1	200	11	4	1	0.014655	0.751954	−2.10563	0.04
A1BG_HUMAN	Alpha-1B-glycoprotein	200	11	4	1	0.381938	0.742658	−1.48566	0.01
KV315_HUMAN	Immunoglobulin kappa variable 3–15	200	11	4	1	0.175186	0.796431	−1.21047	0.005
FETUA_HUMAN	Alpha-2-HS-glycoprotein	200	97	24	1	0.46504	0.201584	0.03433	0.0004
CSF1R_HUMAN	Colony stimulating factor receptor	200	97	24	1	0.107399	0.678371	0.000169	0.00061
A1AT_HUMAN	Alpha-1-antitrypsin	200	97	24	1	0.50398	0.972984	−1.73774	0.0004
HV315_HUMAN	Immunoglobulin heavy variable 3–15	200	0	1	1	0.441371	1.29497	−4.54668	0.00014
A2MG_HUMAN	Alpha-2-macroglobulin	200	37	15	5	0.067335	1.38741	−2.57057	0.00058
TRFE_HUMAN	Serotransferrin	200	50	15	5	0.050994	1.028623	−0.85167	0.00025

a: Protein name identified from Mascot and Uniprot database; b: Fold change obtained among untreated//control and treated Giant cell tumor of bone. Untreated is considered as without any treatment including chemo, radiation, immunotherapy and cryotherapy. Treated group considered as the samples treated using cryoablation, and irradiation.

**Table 2:** Down-regulated proteins identified from untreated/control giant cell tumor of bone compared to cryoablation and irradiation treated.

Accession	Protein Name ^a^	Significance	Coverage (%)	#Peptides	#Unique	GCT_Untreated/Control ^b^	GCT_Cryoablation ^b^	GCT_RAD ^b^	*p*-Value
ENOA_HUMAN	Alpha-enolase	200	50	15	5	−0.18695	1.127769	−0.52723	0.01
FHL1_HUMAN	Four and a half LIM domains protein 1	200	32	12	2	−0.11059	0.930268	−0.54024	0.04
IGHG3_HUMAN	Immunoglobulin heavy constant gamma 3	200	18	3	1	−0.10647	0.956033	−0.22101	0.001
IGHA1_HUMAN	Immunoglobulin heavy constant alpha 1	200	28	13	1	−0.50502	1.027274	−0.39062	0.001
HPT_HUMAN	Haptoglobin	200	29	6	5	−0.58019	0.927425	−0.23422	0.002
PRDX2_HUMAN	Peroxiredoxin-2	200	21	6	6	−0.24551	0.767434	−0.03834	0.01
IGHG2_HUMAN	Immunoglobulin heavy constant gamma 2	200	1	1	1	−0.42138	0.601606	−0.50063	0.0000065
DPYL2_HUMAN	Dihydropyrimidinase-related protein 2	200	1	1	1	−0.53341	0.671534	−0.41069	0.04
FINC_HUMAN	Fibronectin	200	3	2	2	−0.81512	0.7081	−0.23971	0.02
TRFL_HUMAN	Lactotransferrin/Growth-inhibiting protein 12	200	1	1	1	−1.32309	0.707061	−0.04577	0.04
IGJ_HUMAN	Immunoglobulin J chain	200	28	9	5	−0.7484	0.69038	0.149366	0.00032
FLNB_HUMAN	Filamin-B	200	3	2	1	−0.79679	0.708131	0.103967	0.00000561
GDIB_HUMAN	Rab GDP dissociation inhibitor beta	200	11	16	6	−0.63366	0.523302	0.435171	0.01
CATD_HUMAN	Cathepsin D	200	18	2	2	0.137456	0.13756	0.412715	0.0005
KAP0_HUMAN	cAMP-dependent protein kinase type I-alpha regulatory subunit	200	8	2	2	−0.19286	0.249868	0.616559	0.0007
CLIC1_HUMAN	Chloride intracellular channel protein 1	200	13	11	1	−0.38777	−0.32961	0.719213	0.0000024
ALDOA_HUMAN	Fructose-bisphosphate aldolase A/Lung cancer antigen NY-LU-1	200	3	12	1	−0.65764	−0.40178	0.848804	0.003
TPIS_HUMAN	Triosephosphate isomerase	200	7	14	1	−0.82482	−0.31639	0.655647	0.04
PGK1_HUMAN	Phosphoglycerate kinase 1	200	2	16	1	−0.85269	−0.46433	0.613938	0.00012
FIBA_HUMAN	Fibrinogen alpha chain	200	4	1	8	−1.10845	−0.66497	0.757142	0.04
HBAZ_HUMAN	Hemoglobin subunit zeta	200	3	4	2	−0.96093	0.237888	0.116009	0.0023
IGHG4_HUMAN	Immunoglobulin heavy constant gamma 4	200	13	1	1	−1.11525	0.2758	0.094595	0.004
ANXA1_HUMAN	Annexin A1/p35	200	3	1	1	−1.25595	0.194197	0.232402	0.01
AMBP_HUMAN	Protein AMBP	200	3	1	1	−0.00093	0.172501	−2.48943	0.0007
A1AG1_HUMAN	Alpha-1-acid glycoprotein 1	200	23	14	5	−0.59995	0.077871	−0.81155	0.02
S10A9_HUMAN	Protein S100-A9/Migration inhibitory factor-related protein 14	200	3	7	1	−0.73456	0.257952	−0.74316	0.04
PERM_HUMAN	Myeloperoxidase	200	14	1	1	−2.42652	0.801777	−1.24608	0.04
GTR1_HUMAN	Solute carrier family 2, facilitated glucose transporter member 1	200	4	5	1	−1.02121	−0.12839	−0.27789	0.01
TPM3_HUMAN		200	5	16	1	−0.91326	−0.40327	−0.41174	0.005
CAPG_HUMAN	Macrophage-capping protein	200	15	3	1	−1.29189	−1.04556	−3.08074	0.0004
ITIH4_HUMAN	Inter-alpha-trypsin inhibitor heavy chain H4	200	13	12	6	−2.61728	−0.57445	−0.26668	0.00061
FRIL_HUMAN	Ferritin light chain	200	3	7	1	−2.54766	−0.96978	0.245989	0.0004
B3AT_HUMAN	Band 3 anion transport protein	200	3	14	1	−2.00353	0.106701	−0.41339	0.00014
STOM_HUMAN	Erythrocyte band 7 integral membrane protein	200	3	1	4	−2.4425	0.156164	−0.67122	0.00058
AACT_HUMAN	Alpha-1-antichymotrypsin	200	32	6	1	−1.34745	0.192083	−0.0366	0.00025
IGG1_HUMAN	Immunoglobulin gamma-1 heavy chain	200	22	4	1	−1.99553	0.112038	0.194619	0.00014
APOB_HUMAN	Apolipoprotein B-100	200	4	2	2	−1.88271	0.42193	0.588524	0.000056
PEBP1_HUMAN	Phosphatidylethanolamine-binding protein 1	200	8	1	1	−1.92032	0.549155	0.384127	0.00047
CAH2_HUMAN	Carbonic anhydrase 2	200	7	1	1	−1.91215	−0.23354	0.693737	0.00014
GSTP1_HUMAN	Glutathione S-transferase *P*	200	7	1	1	−4.03432	−0.08147	0.859662	0.0056
PPIA_HUMAN	Peptidyl-prolyl cis-trans isomerase A	200	16	2	1	−0.6593	0.020047	0.843733	0.00014
F13A_HUMAN	Coagulation factor XIII A chain	200	16	3	3	−0.77574	0.216645	0.714859	0.00061
SODC_HUMAN	Superoxide dismutase [Cu-Zn]	200	10	2	2	−0.85644	0.197895	0.714553	0.0004
SPTB1_HUMAN	Spectrin beta chain, erythrocytic	200	28	9	5	−0.71009	−0.04636	1.052198	0.00014
HVM17_HUMAN	Ig heavy chain V region MOPC 47A	200	3	2	1	−1.46988	0.246253	1.037756	0.00058
VDAC2_HUMAN	Voltage-dependent anion-selective channel protein 2	200	1	1	1	−1.07957	−0.27273	1.016574	0.00025
HSP7C_HUMAN	Heat shock cognate 71 kDa protein	200	18	2	2	−1.87161	−0.55349	1.177799	0.04
PGS2_HUMAN	Decorin/Bone proteoglycan II	200	18	2	2	−0.37748	−1.37542	1.357619	0.04
VAT1_HUMAN	Synaptic vesicle membrane protein VAT-1 homolog	200	3	1	1	−1.69165	−0.99373	1.390167	0.01
COF1_HUMAN	Cofilin-1	200	3	1	3	−2.07774	−0.98897	1.379402	0.002
IF4A1_HUMAN	Eukaryotic initiation factor 4A-I/ATP-dependent RNA helicase eIF4A-1	200	7	1	1	−1.75171	−1.79621	1.711834	0.0005
NUCL_HUMAN	Nucleolin	200	2	1	1	−2.86071	−2.53729	1.807236	0.003
BIP_HUMAN	Endoplasmic reticulum chaperone BiP/Heat shock protein 70 family protein 5	200	4	1	5	−2.00732	−1.73829	1.556309	0.00061
GANAB_HUMAN	Neutral alpha-glucosidase AB	200	3	1	1	−2.49319	−2.45966	1.608849	0.0004
APOA1_HUMAN	Apolipoprotein A-I	200	13	5	8	−3.31579	−1.46294	1.466519	0.00014
RL18_HUMAN	60S ribosomal protein L18	200	3	1	1	−3.85556	−2.22158	1.512808	0.00058
APOD_HUMAN	Apolipoprotein D	200	28	9	5	−1.7026	−0.60243	1.034735	0.00025
S10A4_HUMAN	Protein S100-A4	200	13	3	1	−1.15342	−1.16094	1.143946	0.04
PRDX6_HUMAN	Peroxiredoxin-6	200	21	4	1	−0.7977	−1.20379	0.928545	0.04
IDHP_HUMAN	Isocitrate dehydrogenase [NADP], mitochondrial	200	18	2	2	−1.7431	−1.2689	1.137871	0.01
RS21_BOVIN	40S ribosomal protein S21	200	8	2	2	−2.49295	−1.47642	1.202521	0.0000006
RS19_HUMAN	40S ribosomal protein S19	200	3	1	1	−2.55879	−1.83901	1.212821	0.04
CO4A_HUMAN	Complement C4-A	200	3	1	1	−2.79494	−1.27031	1.102623	0.04
LEG1_HUMAN	Galectin-1	200	7	1	1	−2.88241	−2.12528	1.046419	0.01
PDIA1_HUMAN	Protein disulfide-isomerase/p55	200	22	4	1	−3.6809	−1.07449	1.012252	0.00061
GELS_HUMAN	Gelsolin	200	4	1	6	−2.06544	−0.18597	1.095377	0.0004
ITB2_HUMAN	Integrin beta-2	200	3	1	1	−1.86435	−0.05573	1.100121	0.00014
C4BPA_HUMAN	C4b-binding protein alpha chain	200	3	1	1	−2.41077	0.128627	1.05217	0.00058
AOC3_HUMAN	Membrane primary amine oxidase	200	3	1	1	−2.95289	0.051086	0.980408	0.00025
HBA_HUMAN	Hemoglobin subunit alpha	200	3	1	2	−3.05928	−0.02117	1.105323	0.001

a: Protein name identified from Mascot and Uniprot database. b: Fold change obtained among untreated/control and treated Giant cell tumor of bone. Untreated/control is considered as without any treatment including chemo, radiation, immunotherapy and cryotherapy. Treated group considered as the samples treated using cryoablation and irradiation.

**Table 3:** Demographic data of giant cell tumor patients for comparative proteomic analysis.

Patient	Sex	Age	Location	Size of Tumor	Treatment	Reconstruction	Local Recurrence	Chemo & Radiation
1	F	33	Distal femur	8	Wide excision	recycle autograft	Yes	No
2	M	36	Proximal humerus	5	Curettage + cryoablation	recycle autograft	No	No
3	M	45	Proximal humerus	7	Wide excision	recycle autograft	Yes	No
4	F	55	Distal femur	18	Curettage + cryoablation	recycle autograft	No	No
5	F	46	Humeral shaft	4	Wide excision	recycle autograft	Yes	No
6	F	54	Distal femur	6	Curettage + cryoablation	recycle autograft	No	No
7	M	47	Acetabulum	5.6	Wide excision	bone grafting	Yes	No
8	M	58	Humeral Shaft	7	Wide excision	bone grafting	Yes	No
9	F	38	Acetabulum	6	Curettage + cryoablation	bone grafting	No	No
10	M	63	Distal femur	18	Curettage + cryoablation	total hip arthroplasty	No	No
11	F	64	Proximal humerus	10	Wide excision	bone grafting	Yes	No
12	M	56	Distal femur	12	Curettage + cryoablation	bone grafting	No	No

## Supplementary Materials

The following supplementary information is provided with this manuscript: Table S1: A representative xl-data sheet of untreated/control Giant Cell Tumor of Bone mass spectrometric analysis; Table S2: A representative xl-data sheet cryoablation treated Giant Cell Tumor of Bone (GCT_Cryoablation) mass spectrometric analysis; Table S3: A representative xl-data sheet irradiation treated Giant Cell Tumor of Bone (GCT_Irradiation) mass spectrometric analysis Table S4: Comparative proteomic evaluations of GCT-untreated/control vs. GCT-treated with cryoablation and irradiation.
